# Mr. Borrett *on Gastritis and Hydrophobia*

**Published:** 1814-01

**Authors:** G. Borrett

**Affiliations:** Great Yarmouth


THE
### Medical and Physical Journal.

1 OF VOL. XXXI.] JANUARY, 1814. [NO. 179.

"For many fortunate discoveries in medicine, and for the detection of nume-
"rous errors, the world is indebted to the rapid circulation of Monthly
"Journals; and there never existed any work to which the faculty in
"EUROPE and AMERICA were under deeper obligations than to the
"Medical and Physical Journal of London, now forming a long, but an
"invaluable series."—RUSH.

### *To the Editor of the Medical and Physical Journal.*

SIR,

PERMIT me, through your valuable Journal, to return my best thanks to Mr. Abel of Halesworth, for his paper in your Journal of last August, claiming for me, in answer to Dr. Kinglake, the priority of suggesting a practice in hydrophobia, founded on a similarity between this disease and gastritis; and to correct the typographical error in his name, which is Abel and not Aber. This gives me an opportunity of acknowledging Dr. Kinglake's candid reply to Mr. Abel; and also to notice an error of the press in Dr. Kinglake's paper, where Barrett occurs for Borrett.

I must apologize to Dr. Adams for having been prevented by my engagements from paying earlier attention to his paper, by which he has given us to understand, that in his second edition of Morbid Poisons, page 380, he had anticipated the similarity of disease between gastritis and hydrophobia, and there recommended the *Auxilium anceps* of Celsus to be pushed much further than hitherto attempted. The doctor thus deprives me of the little merit to which I might have considered myself before entitled, of contrasting the two diseases, and grounding, upon the positive existence of inflammation of the stomach in hydrophobia, (which inflammation was discovered by examination, and, perhaps, was not known by him to exist when he wrote upon Morbid Poisons, and which indeed he now seems to doubt) the modus medendi, bleedings ad deliquium animi. Thus, in the moments of speculative fancy, he takes from me the reward of hours of labor: is not this very hard, that he, while soaring in these light airy regions, should, like the kite, pounce down upon his prey, and betray his rapacity?

I have only Dr. Adams's first edition of Morbid Poisons, and therefore have not been able to see whether, in his latter work, he has directed the mind to the similarity of the two diseases, by comparing the symptoms which take place in hydrophobia with those of gastritis, viz. the great pain in the hypogastric region, particularly under the cartilago ensiformis; the small, hard, and quick pulse, sometimes intermitting; the great prostration of strength in a few hours, so very remarkable in both diseases; painful hiccough; and that liquids, if they are swallowed, are as generally vomited, while the features of the sufferers in both diseases are strikingly alike distressing, &c. &c.

If, by thus contrasting the two diseases, the *Auxilium anceps* of Celsus had followed as the only remedy, I would readily have admitted Dr. Adams's claim to priority in recommending bleeding as the only remedy in hydrophobia; but it is not merely bleeding,—*it is the time, the extent, and the manner, in which the operation is performed.*

Dr. Adams having laid claim to this, the most important part of my paper, in my estimation, published March 14, 1809, vol. xxi. of the Medical and Physical Journal, begins his animadversions upon it, by doubting the presence of inflammation of the stomach in the case of Elizabeth Kirk, as well as in that of Dr. Pinckard's, &c. because Mr. Hunter's account, he says, of the digestion of the stomach after death, accords with most of the appearances described by us as inflammation of the stomach in hydrophobia and its consequences.

I have not Mr. Hunter's Animal Economy, 2d edition, to which Dr. Adams refers me, and I think if I had, I should not be induced by its perusal to attribute, with him, the appearances of the stomach described in my case of hydrophobia *to digestion* after death, rather than inflammation, as Dr. A. would willingly have me believe, for the following reasons:—Whoever will refer to my case, or the quotation from it by Dr. A. will observe, that the parts described as particularly inflamed are the *œsophagus at its cardiac extremity, the cardia, and for the breadth of the palm of the hand, more particularly around the cardia,* and extending towards the large end of the stomach. By this description I meant to confine the appearances of inflammation to the cardiac portion of the stomach and its smaller curvature, according to its natural situation, to the upper part of the stomach, and not the large end and curvature, for there the marks of inflammation, if any, were very faint. It must be evident that the solution of the stomach by its *gastric fluid* after death must be greatest at the most depending part, where

 the

*the fluid* would rest; and this would be about the larger curvature, and great end of the stomach, and which is so expressed by Dr. Adams's quotation from Mr. Hunter's Animal Economy; but at this very part, the appearances of the stomach in Elizabeth Kirk's case were natural, or nearly so.

I am much obliged to Dr. A. for his long quotation from Mr. Hunter, by which I learn that "there are few dead bodies in which the stomach at its great end is not in some degree digested, and one who is acquainted with dissection can easily trace the gradations." The doctor goes on to remark, "these gradations depend very much on the season of the year, and the mode of dying. If in the summer, the patient being in previous high health, and killed by violence soon after a meal, the substance of the stomach is sometimes *completely* eroded; but this is rare." Indeed I think it is rare, if I may judge from the following solitary instance to the contrary. The convict Hannah, aged 64, of athletic stature, was executed at Yarmouth on Monday, Sept. 6th, 1813, at twelve o'clock at noon, for the murder of his wife, and his body given to the surgeons for dissection. He had been in prison from the 15th of April, on which day the murder was committed. He was in full health and spirits, for he seemed not to apprehend the danger of his situation (not expecting death); and was supplied by his relations until the day of his condemnation, Friday the 3d preceding his execution, with a bottle of porter per day, and plenty of meat. From the Friday that he received his sentence, until he was executed on the Monday (three days), he had one pound of white bread per day, and water, which bread he regularly ate, and a part even on the morning of his execution, so that we have here a case exactly in point, *a man in good health, killed by violence, in hot weather, and after a meal.* The body was submitted to dissection the following morning (Tuesday), and on the Wednesday morning, eight o'clock, forty-four hours after he was executed, the abdominal cavity was opened; the stomach, and part of the œsophagus and duodenum were removed from the body, and carefully and minutely examined, as it was expected that the stomach would be found *completely* eroded, at least somewhat digested at its large end and larger curvature, as Dr. Adams represents; but after a most diligent search with several of my surgical friends (three of whom had lately been in the habits of constant dissection), not the smallest abrasion of the villous coat, or "altered appearance of the inner surface of the great end of the stomach, with the other parts of its inner surface," could be discovered; nor certainly were there any of the diseased appearances described by me in the stomach from hydrophobia.

The stomach of Hannah showed a regular arterial congestion on its inner surface, more marked towards the pylorus, in the course of the different branches, given off by its arteries the gastro-epiploic. Thus it is manifest that digestion of the stomach, under the most favored circumstances, does not always take place: how far it generally does, is much to be doubted. Why, therefore, let me ask Dr. Adams, should it generally take place (which we are led to conclude from his paper) in cases of death from hydrophobia? Had the diseased appearances in the stomach, described by me, taken place in consequence of digestion, surely that digestion would not have occupied the cardia, and extended up two inches of the œsophagus, any more than in the case of Dr. Pinckard's, so accurately and well delineated, and who describes the whole of the pharynx and œsophagus in a state of inflammation, and says, "at the lower part of the œsophagus, about half an inch from the cardiac orifice of the stomach, was an eroded spot, nearly the size of a shilling, assuming an appearance as if the inner coat had been separated, and shrivelled up by scorching. A few inches below the cardia was a fulness of the vessels of the villous coat, which caused a spotted and circumscribed redness about three or four inches in diameter." In this case, is it not probable, nay certain, according to the doctor's own statement, if these appearances had been occasioned by the action of the *gastric fluid*, that they would have occupied the great end and larger curvature of the stomach, rather than rising up into the œsophagus? When Dr. A. has seen a case of fatal hydrophobia, and dissected it, we shall meet upon the same ground: until then I must support Dr. Pinckard's case, and maintain my own opinions, that we are nearer the truth when I assert, that in nine cases out of ten of fatal hydrophobia, the stomach or œsophagus, or both, will be found upon dissection the *principal* seat of the disease, and to have undergone inflammation, and that early bleedings ad deliquium is the only *rational*, and I may *now* say *known remedy*.

Whoever will take the trouble to read Dr. Shoolbred's successful case, published at Calcutta, May 18, 1812, will have his attention rivetted to the principal seat of the disease by the detail of symptoms. He says, "He (Amier the patient) was exceedingly impatient of restraint, and whenever he could get a hand disengaged, he immediately struck the pit of his stomach with it, pointing out that part as the seat of some indescribable uneasiness." That uneasiness was was removed by copious bleeding, but before the second bleeding, Dr. Shoolbred states that the pain had returned, as follows:—" He (Amier) also put his hand to the region of the stomach, and said, that the pain in that part was returning." Are we not to believe in the feelings of our patients, when, too, dissections confirm and support the seat and truth of these diseased sensations?

Dr. Adams goes on to conjecture " that the hydrophobic poison may, like some other poisons, be determined in its influence to particular parts, but not with the same certainty, to the same parts in that order," with many other judicious remarks on the different poisons of diseases, as the variolous, varicellous, scarlatina, venereal; the atmospheric poisons, as influenza, dysentery, &c. But the great diversity of the parts affected by the canine poison, and the different symptoms occasioned by it, which, according to the conjectures of the doctor, exceed all other morbid poisons, have plunged me, I confess, into as great a dilemna, as I recollect to have experienced on the subject of *small-pox*, when the doctor, some time ago, described the *pearl small-pox*, in which, he will pardon me when I confess, I had no faith.

However, the doctor has admitted that rabies canina is an inflammatory disease; that this inflammatory diathesis being set up in the system by the poison, is more particularly directed to the different mucous membranes (or secreting membranes), so that we are led by sympathies from the nose to the urethra, from the mouth to the anus, bewildered in the examination of the encephalon, in which there is no mucous membrane,—and so distracted in our senses by the great variety of the parts affected, that we shall scarcely know, after a few more observations on this intricate subject, whether the œsophagus, the stomach, the nose, the urethra, or the anus, stands first in the order of parts diseased; thus doubting every thing we can fix on nothing, and the little gleam of light which seemed breaking in upon us will be extinguished amidst the darkness of conjecture.

There are, on all subjects and sciences, facts or first principles, which, if we lose sight of, our labors are but vain. I must now beg leave to offer my best thanks to the doctor for his paper on hydrophobia; and to assure him, that if any sentence in this reply should be found to carry with it an unfriendly construction, or too much personality, I shall be sorry, and beg for sincere pardon.

I am, Sir,
Your obedient humble Servant,
G. BORRETT.

*Great Yarmouth,*

